# Exploring the nexus between physical environments, resident preferences, and usage frequency of community centers: Evidence from two Korean neighborhoods in Seoul

**DOI:** 10.1371/journal.pone.0295530

**Published:** 2023-12-14

**Authors:** Chia-Yuan Yu, Ayoung Woo

**Affiliations:** 1 School of Public Administration, College of Community Innovation and Education, University of Central Florida, Orlando, Florida, United States of America; 2 Graduate School of Urban Studies, Hanyang University, Seoul, South Korea; University of Hong Kong, HONG KONG

## Abstract

Community centers offer a public space for physical activities, attracting residents with diverse interests and abilities, and promoting social interaction and connection, which in turn enhances individuals’ physical and mental fitness and fosters a sense of community. When exploring the correlation between community space accessibility and usage frequency, it is crucial to consider empirical evidence and resident preferences. Nevertheless, the role of physical environments in determining community center usage frequency, while also considering residents’ inclination for effortless access, remains unclear. This study investigated the impact of resident preferences and satisfaction with the accessibility of community centers and physical environments on usage frequency, addressing a gap in previous research. Structural equation models were utilized to analyze a household drop-off survey consisting of 186 residents gathered from two neighborhoods in Korea. The results revealed that while the preference for easy access to community centers did not have a substantial effect on their usage frequency, satisfaction with easy access to such centers was positively linked to usage frequency. Furthermore, the perception of physical environments as being walkable and having a diverse range of amenities was associated with a higher frequency of community center usage. These findings have policy implications as they can help increase the usage frequency of community centers and enhance overall well-being in regenerated neighborhoods.

## Introduction

Urban regeneration has gained importance for policymakers and planners seeking to revitalize neglected neighborhoods. In such projects, community centers are established to provide public spaces for residents, fostering social engagement and capital. However, the effective construction and placement of these centers in regenerated communities is a topic of interest, as policymakers and planners aim to promote resident utilization of these spaces, ultimately enhancing social integration and strengthening social capital in these neighborhoods [1, 2].

Urban regeneration in Korea differs from large-scale redevelopment efforts. While urban redevelopment can displace tenants and erode community heritage, collaborative urban regeneration projects involving the government and residents prioritize neighborhood revitalization, heritage preservation, and resident involvement [3]. Community centers play a central role in these initiatives, fostering community engagement and empowerment. Notably, in Korea’s urban regeneration projects, community centers are distinct as they are solely established and funded by the public sector (e.g., Seoul Metropolitan Government), with residents having significant control over their operation and management [2]. Community centers, central to community life, thrive on resident involvement in their design, operation, and management, typically through Local Community Committees (LCCs). However, regeneration efforts have sparked concerns about low community center usage in these neighborhoods, prompting calls for effective strategies. Location, especially accessibility, has emerged as a key concern. Given the growing focus on the location-usage relationship, empirical evidence and residents’ access preferences should be considered. Additionally, the impact of physical environments on usage frequency while aligning with residents’ access preferences remains uncertain.

This study focuses on the following research questions: Do residents experience disparities between their preferences and their satisfaction with access to the community center? Does the preference for easy access directly or indirectly affect center usage frequency? Do physical environments impact center usage after accounting for access preferences? Using structural equation modeling, the study explores how residents’ preferences and satisfaction with community center accessibility affect usage, considering physical environments. These insights benefit urban planners, policymakers, and researchers globally seeking to enhance community center use and sustainability in regeneration projects.

## Literature review

### Characteristics and concerns of community centers in regenerated neighborhoods

In 1929, Clarence Perry introduced a groundbreaking urban planning concept known as the “neighborhood unit,” aimed at creating self-contained, functional neighborhoods within metropolitan areas. At the core of this innovative model lay a fundamental principle: strategically situating the neighborhood’s central hub, typically represented by the school, to ensure that students could conveniently live within a half-mile, easily walkable distance from their educational institution [4]. Perry’s vision also encompassed the strategic placement of commercial zones and high-speed roadways, such as highways and arterials, around the periphery of the neighborhood [4]. Following this innovative approach, the perception of where and how community centers should be situated and utilized underwent a significant transformation within the sphere of urban planning theory and practice. This concept has notably influenced urban planning in South Korea, where community centers have been strategically positioned as central hubs within neighborhoods.

South Korea has substantially used a large-scale urban redevelopment to rebuild neighborhoods since the 1980s, which have resulted in positive outcomes such as improved economic conditions and more housing units [3]. However, these approaches have also been criticized for displacing residents and losing of social assets [5–7]. Hence, during the decade spanning from 2010 to 2019, Korea transitioned toward a more grassroots-oriented strategy, fostering citizen involvement in initiatives focused on revitalizing urban areas [3]. This strategy enables community members to articulate their perspectives and exert meaningful influence on the planning process. However, the implementation of this approach faces challenges, particularly in providing public support for residents such that they can actively participate in the planning process [8]. Despite these challenges, planners and scholars have widely regarded urban regeneration initiatives as successful in revitalizing blighted communities and promoting community engagement, partly because of the active involvement of local residents in the planning and execution of these projects.

The Residential Environment Improvement Project (REIP) is a prominent urban regeneration mechanism in Korea that focuses on rejuvenating both the physical and social aspects of residential areas [9]. Since its inception within the realm of urban regeneration strategies, the REIP has evolved into one of the most significant tools for revitalizing deteriorated and distressed neighborhoods in Korea. Despite being characterized as a smaller-scale endeavor in comparison to alternative redevelopment approaches, the REIP places a distinct emphasis on the preservation and enhancement of social assets within communities (Seoul Metropolitan Government, 2021). Distinct from other urban redevelopment endeavors that typically entail the demolition of aging structures, the REIP emphasizes preserving and enhancing social assets while improving infrastructure to revitalize distressed neighborhoods [3, 10]. Regarding physical regeneration, the REIP encompasses the revitalization of decayed infrastructure and built environments, encompassing streets, water and sewage networks, street CCTV cameras, and various other elements, to rejuvenate blighted neighborhoods. Moreover, along with physical transformation, the essence of the REIP lies in the preservation and augmentation of social capital within the community (Seoul Metropolitan Government, 2021). Consequently, the provision of community centers, where residents can engage in social interactions and activities, has emerged as a pivotal consideration for planners and policymakers in achieving the regeneration of REIP sites. The REIP’s approach to urban regeneration represents a shift towards a more holistic approach that recognizes the importance of preserving each community’s unique social characteristics and needs. The project’s emphasis on revitalizing the physical and social dimensions of communities is widely viewed as a more sustainable approach to urban regeneration that can enhance community well-being.

At locations where the REIP is put into effect, the community center plays a crucial role in facilitating various community events and fostering social connections among residents. Residents proposed the need for indoor and outdoor attributes of community centers built by the government. At the beginning of the regeneration initiative, community members worked alongside planners and public officials to identify essential upgrades to residential environments and community spaces. Following the agreement, the responsibility of acquiring land and constructing the center fell on the Seoul Metropolitan Government [9]. Community centers at REIP sites stand out from conventional centers run by the public sector, as they are established and managed entirely by the residents themselves [2]. Whereas the public sector offers physical infrastructure, community centers encourage residents to participate in community activities, including neighborhood meetings, cleaning initiatives, singing lessons, preschool childcare, knitting clubs, film screenings, and community festivals. By enabling the establishment and operation of community centers, policymakers and planners acknowledge that the REIP can provide residents with stable physical and economic resources to participate in diverse events, thereby fostering social cohesion and revitalizing the community. This perspective underscores the significance of the community center in the REIP project, setting it apart from other urban redevelopment endeavors.

The allocation of substantial financial supplies to establish community centers at REIP sites reflects the recognition of planners and public officials regarding the potential benefits they can offer. According to the Seoul Metropolitan Government (9), by 2022, the implementation of 86 REIPs was completed, and for Seoul REIPs, 62.5% of the total expenses were allocated to building community centers for residents. On average, each site received a budget of 1.9 million USD to establish these centers. In contrast, the introduction of community centers has faced resistance from some residents who question their efficacy [8]. After the completion of the REIPs, there has been a growing concern about the low frequency of community center usage in neighborhoods. Many believe that community centers are underutilized because their site locations do not align with the opinions of residents. For instance, community centers are often located in less accessible areas because these areas tend to have more affordable land prices for the public sector. Therefore, some residents advocate reallocating REIP budgets from community center construction to urban redevelopment strategies, such as replacing distressed buildings with new facilities, for more effective outcomes, such as increased individual property values [8]. Despite the challenges posed by the low frequency of community center usage, several planners, researchers, and administrators remain supportive of their potential to promote events and gatherings and improve social capital through regeneration initiatives [11, 12]. This ongoing support demonstrates the continued importance of community spaces in the REIP as a tool for sustainable urban regeneration. However, the provision of community centers may not support the notion that residents would naturally use these public facilities. There is limited empirical evidence on encouraging residents to use community centers that potentially enhance social capital in Korea’s regeneration project settings. Hence, this study fills a gap by empirically investigating how residents’ preferences and satisfaction with the accessibility of community centers influence the frequency of their use, while also accounting for the physical environments in neighborhoods.

### Factors associated with community center use

Community centers are commonly created as a communal area where residents can socialize, interact with one another, and participate in diverse community events and programs [13]. These spaces are typically established by public or private organizations with the objective of fostering community development and enhancing the well-being of local inhabitants [11]. The utilization of a community center may be subject to a diverse range of factors that can potentially affect it.

In terms of socio-demographic characteristics, individual age influences community center use, with older adults using these facilities more frequently than younger age groups [14]. This can potentially be attributed to various factors, such as social isolation, limited mobility, and retirement, which can increase the necessity for social interaction and community engagement. Consequently, community centers can provide valuable resources for older adults to meet their social and recreational needs. Gender also plays a role in the frequency of community center use [15]. For example, work or career demands may limit male access to community centers. Additionally, some community center activities may be perceived as gender-specific, which could impact usage patterns. Therefore, it is vital to understand gender differences in community center use when designing inclusive programs and activities. High-income households may use community centers less frequently because of their access to other amenities or facilities that offer similar services, such as private gyms or clubs, which may be better suited to their preferences [16]. Additionally, high-income individuals may have busy work schedules that limit their leisure time. In terms of marital status, unmarried individuals may have fewer social connections and support systems, potentially making them less likely to use community centers [17]. Conversely, households with more children may use community centers more often due to the range of programs and services available that cater to children, such as after-school care, summer camps, and youth sports leagues [18]. This makes community centers a convenient and affordable option for meeting childcare needs, while providing children with opportunities for socialization and physical activity. Furthermore, community centers may offer programs and services designed to support families, which could encourage households with more children to use these facilities regularly.

The use of a community center can be significantly influenced by the surrounding physical environment [19–22]. Factors such as walkability, access to transit, and amenities such as parks and recreational facilities can contribute to the likelihood of individuals using a center by providing convenient and accessible options for transportation and recreation. For instance, people residing in areas with high walkability or easy access to public transportation are more likely to visit facilities [19]. Safety is also a critical factor influencing the use of community center. A perception of safety concerns or crime in the surrounding area can discourage individuals from using the center, particularly during evening or night hours [21]. Additionally, cleanliness around the community center is essential for creating an inviting and welcoming space for individuals to visit [22].

The indoor and outdoor attributes of a community center, such as its size, cleanliness, and safety, can have a significant impact on its use [23]. The size of a community center is an important factor, as it determines the capacity and range of activities that can be offered. A larger community center can accommodate a wider range of events and activities, attracting more individuals to use the facility [24]. A clean and well-maintained community center can improve user experience and encourage individuals to visit and use the center more frequently [24]. A perception of safety concerns or incidents in the indoor and outdoor environment can discourage individuals from using the facility [25]. By implementing appropriate safety measures, such as hiring trained personnel, installing security cameras, and developing emergency response plans, it is possible to enhance the safety of the environment and encourage more people to use the center.

The use of community centers can be influenced by program attributes, such as education, leisure, childcare, and free-sharing programs [26]. Offering a diverse range of programs that cater to various interests and needs can attract a wider audience and increase the likelihood of their use [27]. The quality and variety of programs offered are critical factors that influence the use of community centers. Centers that provide high-quality programs led by qualified instructors can significantly enhance user experience and encourage individuals to return to the facility [27]. Therefore, prioritizing the development and implementation of diverse and high-quality programs can help community centers increase their use and appeal to a broader audience.

Accessibility is another key factor that may affect community center use. Accessibility to community centers not only promotes physical activity but also increases the use of community centers among individuals [28]. When community centers are easily accessible to individuals, they are more likely to visit and utilize the available facilities because of the convenience of their location, making it easier for residents to access the center and participate in activities [29]. This, in turn, can lead to increased social interactions and a sense of community, which have been linked to positive health outcomes. Community centers with easy access to public transportation, bicycle lanes, or walking paths are more heavily utilized by residents. Therefore, policymakers and urban planners should consider the location and design of community centers to increase accessibility and promote use among individuals, which, in turn, can lead to various health and social benefits.

### Neighborhood self-selection

Neighborhood self-selection refers to the process by which individuals choose to reside in areas that provide resources and amenities aligned with their desired lifestyles. This phenomenon has been extensively studied to investigate the associations between physical environment and transportation choices [30]. Self-selection has also been recognized as pivotal in investigating the association of built environments on physical activity [31]. Extensive research on residential mobility and housing selection has investigated the significant effects of race/ethnicity and income on the process of self-selection [32]. Self-selection in neighborhoods with similar demographics can result in the segregation of different racial and ethnic groups across neighborhoods. If low-income households select neighborhoods based only on affordable housing, and if these communities do not have sufficient resources for physical activity, the association between the built environment and levels of physical activity may be overestimated. Thus, various factors can influence physical activity, including the physical environment, predisposition to physical activity, and variables associated with physical activity that influence residential selection, or a combination of these variables [31].

Furthermore, this process can pose difficulties in distinguishing the impact of environmental features on behavioral outcomes from an individual’s preference for features that support their preferred behaviors [33]. For example, when examining the relationship between proximity to community centers and frequency of use, an individual’s desire to spend time in the community center may be the driving force behind their decision to live near the facility. Consequently, this preference may serve as an indicator of behavior [33]. Thus, individuals who prioritize using community centers may opt to reside in places that offer convenient access to such facilities, and this preference for accessibility can affect their frequency of use, independent of the physical environment. If the phenomenon of neighborhood self-selection is not taken into account, it may lead to an inaccurate assessment of the effect of neighborhood environmental characteristics on behavioral outcomes. Thus, it is crucial to consider this bias when evaluating the impact of physical environments on behavioral outcomes to avoid misleading conclusions and ensure that policies are based on accurate assessments.

When examining the relationship between accessibility to community spaces and frequency of use, it is essential to consider both the physical environment and residents’ preferences. However, our understanding of whether people use community centers solely because of physical environments, preference/self-selection, or a combination of both is limited. A few studies have explored the connection between preferences, satisfaction with community centers, and usage frequency. Additionally, accessibility to community centers may moderate the relationship between self-selection/preference for accessibility to community centers and usage frequency. In other words, individuals who prioritize utilizing community centers are positively correlated with residing in areas that offer convenient access to these facilities, thereby increasing the probability of their usage. These findings could aid policymakers and urban planners in devising strategies to improve community resources accessibility, encourage physical exercise, and advance communal welfare.

## Materials and methods

The study sought to respond to these three research inquiries:

- Research question 1: Do residents experience disparities between their preferences and their satisfaction with access to the community center?
- Research question 2: Does the preference for easy access to the community center (through access from their residence) have a direct or indirect impact on the frequency of use of the community center?
- Research question 3: Does physical environment have a significant impact on the frequency of use of community centers after considering the preference for easy access to community centers?

Gaining insights into how self-selection/preference and physical environments affect the frequency of use of community centers will enable us to develop better policy recommendations. These recommendations could focus on improving access to community centers and investing in the surrounding environment to promote greater usage. The goal of these efforts is to ensure that the center is responsive to the needs and preferences of the residents it serves.

**Fig 1:**
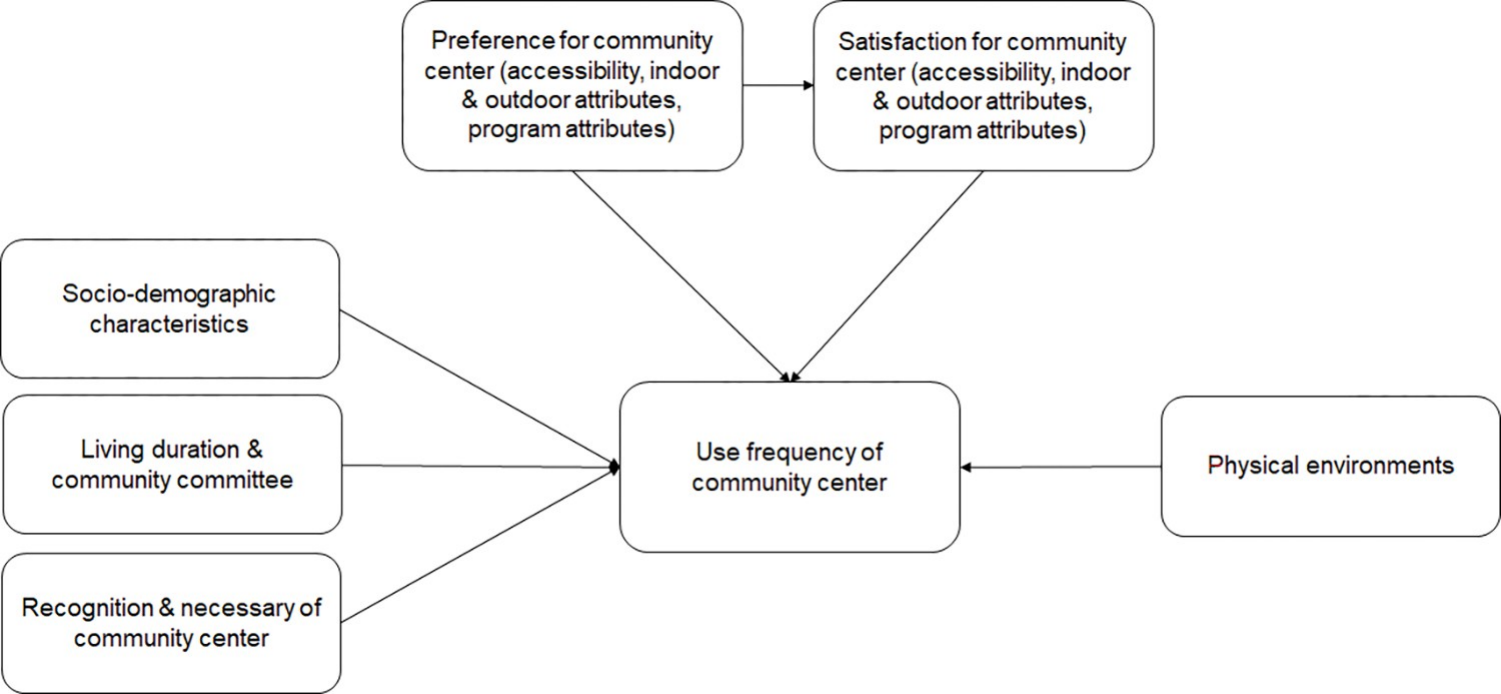
Conceptual framework.

The conceptual framework of this study (Fig 1) was developed through a literature review and considered various factors that may influence the frequency of community center use. These factors include socio-demographic characteristics, duration of residence, community involvement, perception of the importance of community centers, preferences and satisfaction with the community center, and built environments. The preference for the community center is linked to the frequency of use both directly and indirectly through satisfaction with the community center.

### Study area, population, and data

The objective of the REIP is to enhance the built and social fabric of disadvantaged neighborhoods. From 2012 to 2020, 67 deteriorated neighborhoods in Seoul were identified for regeneration using the REIP. Twenty-eight of these neighborhoods have community centers built to enhance the social environment and revitalize the area, while the remaining 39 neighborhoods are undergoing infrastructure improvements and construction of new community centers to further enhance the quality of life in these areas [9].

**Fig 2:**
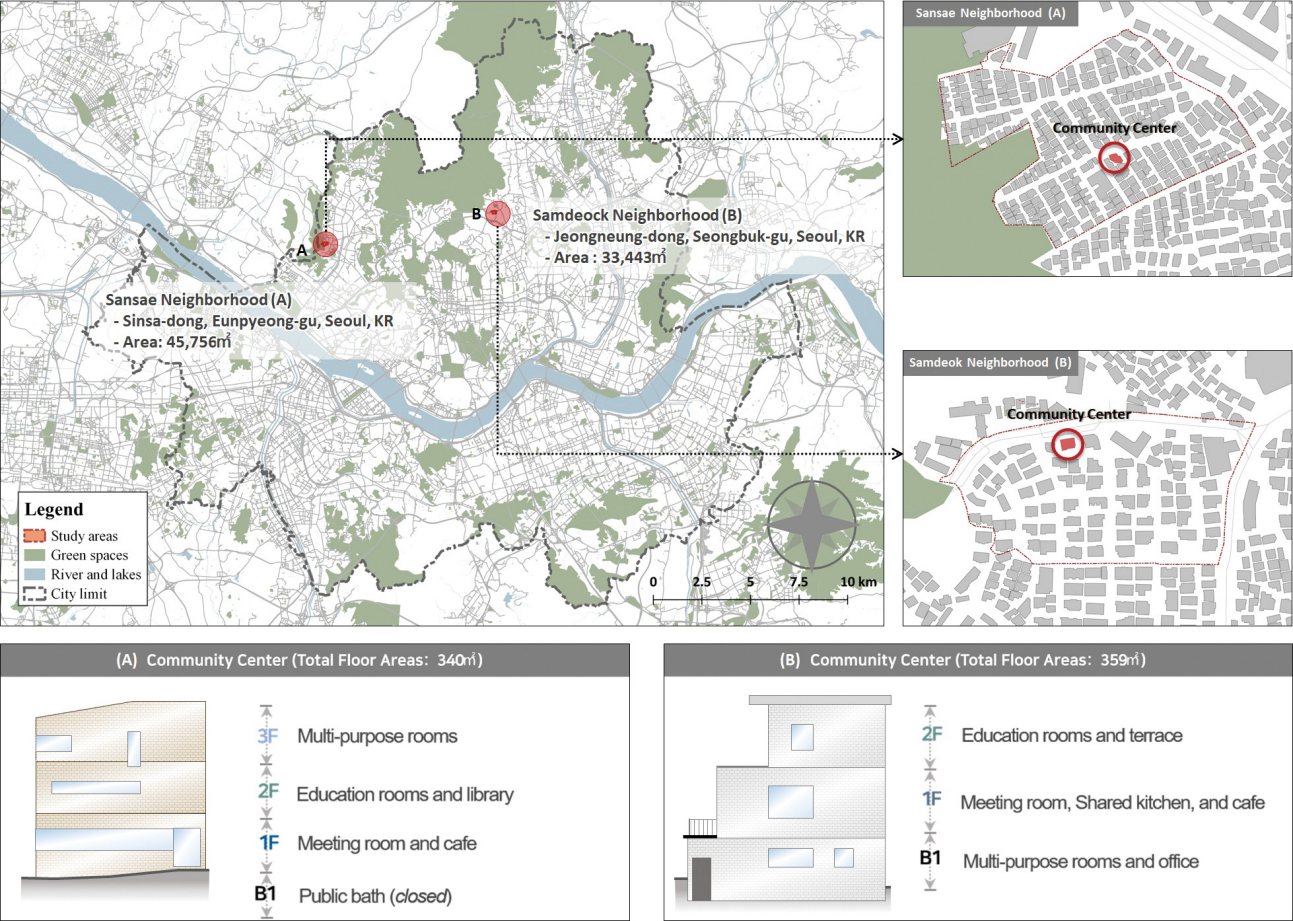
Examples of community centers located in the Sansae and Samdeok of Seoul.

Two neighborhoods that were completed by the REIP were selected for the study: Sansae and Samdeok neighborhoods. Sansae and Samdeok are situated approximately five kilometers northwest and northeast of downtown Seoul, respectively. In 2012 and 2013, Seoul introduced the REIP in these two neighborhoods as the initial phase of regenerating deteriorated areas. With the aim of achieving social regeneration, the Seoul Metropolitan Government constructed community centers in both Sansae and Samdeok in 2014 and 2015, respectively, alongside the revitalization of the physical environments (Seoul Metropolitan Government, 2021). These two neighborhoods, in particular, faced a notable deficiency in community hubs; there was a conspicuous absence of community spaces in the neighborhoods, such as schools, religious institutions, and social welfare facilities, where residents could actively participate in social interactions and engage in various activities. However, after the construction of community centers in the neighborhoods, both community spaces have been consistently operated by residents, distinguishing them from other REIP sites. Additionally, these neighborhoods share similarities in terms of their community center background and characteristics (Fig 2). Since the implementation of the REIP, these two neighborhoods have emerged as demonstration projects and representative examples of successful REIP regenerations in Seoul. Consequently, these two neighborhoods are suitable for the study, given that the consistent operations of their community centers serve as successful REIP models, ensuring the mitigation of short-term fluctuations and distortions in conducting the analyses.

To provide residents with community spaces that were previously unavailable, community centers were built in the neighborhoods of Sansae and Samdeok, with the Seoul Metropolitan Government overseeing the construction. Residents were provided financial assistance by the public sector to facilitate the coordination and management of the centers [9]. Although Sansae has a larger population than Samdeok, both neighborhoods have similarities in terms of environmental conditions and REIP planning. The study area had previously dilapidated housing units and deteriorated infrastructure, raising safety concerns about crime (Seoul Metropolitan Government, 2021). The two centers are also similar in terms of their total floor area, construction costs, and programs initiated and run by the residents themselves. Both neighborhoods aimed to enhance their deteriorating conditions by promoting environmental sustainability through various measures, such as creating pedestrian-friendly streets, ensuring access to efficient water and sewage systems, and ensuring safety from crime. These enhancements aimed to encourage walking behavior among residents, promote in-person communication, and strengthen social connections. These communities offer an appropriate setting to examine the influence of community center utilization on residents, as they reduce the impact of unobserved factors such as the size, condition, and duration of center operation, as well as the revitalization of the surrounding environments. The achievement of these neighborhoods in improving social well-being with the aid of community centers has inspired numerous other REIPs to follow a similar approach. Nevertheless, there is a dearth of empirical evidence on the notion of residents’ utilization of community centers.

This study employed a survey to investigate the frequency of community center utilization among adults aged 19 years and above in the Sansae and Samdeok neighborhoods. This survey evaluated socio-demographic characteristics, preferences and satisfaction with community centers, perceptions of physical environments, and the frequency of utilizing community centers. It was conducted by a professional survey firm (Korea Research Corporation) by request of the Seoul Metropolitan Government in 2022, stratified by two neighborhoods, and one adult per household was interviewed in-person after providing them with the survey questionnaire. The final sample size was 168 respondents. This survey was confirmed exemption by the members of the Institutional Review Board (IRB) of Hanyang University (No. HYUIRB202203005). The raw data of survey variables and the data sets are provided in the S1 Data.

### Variables and measurements

Table 1 presents a list of variables and their measurements. This study investigated the frequency of community center utilization by requesting respondents to rate their frequency of use on a scale of 1 to 5, with 1 indicating “not often” and 5 indicating “very often”.

**Table 1:** List of variables, measurements, and their descriptive statistics.

Domain	Variable	Measurements	Descriptive statistics	
			Mean (SDa) or % of "1" for binary variables	Minimum-Maximum
**Dependent Variable**	Use frequency of community center	How often do you use the community center? (Likert scale: very often = 5)	1.67 (1.12)	1–5
**Socio-demographic characteristics**	Age	Respondent’s age (years)	54.99 (10.59)	19–80
	Gender	Gender (0 = female; 1 = male)	26.76%	0–1
	Income	Household monthly income (1 = below 500,000 KRWb; 2 = 500,000–999,999 KRW; 3 = 1,000,000–1,499,999 KRW; 4 = 1,500,000–1,999,999 KRW; 5 = 2,000,000–2,499,999 KRW; 6 = 2,500,000–2,999,999 KRW; 7 = 3,000,000–3,499,999 KRW; 8 = 3,500,000–3,999,999 KRW; 9 = 4,000,000–4,499,999 KRW; 10> = 4,500,000 KRW)	7.56 (2.40)	2–10
	Marital status	Marital status (0 = other status; 1 = single)	1.54%	0–1
	Homeownership	Homeownership (0 = no; 1 = yes)	53.86%	0–1
	Education level	Education level (1 = elementary; 2 = middle; 3 = high; 4 = university; 5 = graduate)	2.89 (0.76)	1–4
	Number of household members	Number of household members (continuous)	3.17 (0.91)	1–5
	Number of children	Number of children (continuous)	1.78 (0.58)	1–5
	Community area	0 = Samdeok community; 1 = Sansae community	71.53%	0–1
**Living duration & community committee**	Living duration	Total time spent residing in the area (months)	165.46 (88.04)	12–487
	Community committee	Are you currently a member of the Local Community Committee (LCC)? (0 = no; 1 = yes)	2.91%	0–1
**Recognition & necessary of community center**	Recognition of community center	Do you know that there is a community center in your town? (0 = no; 1 = yes)	66.72%	0–1
	Necessary of community center	Do you think the community center is necessary in the town? (0 = no; 1 = yes)	43.34%	0–1
**Preference for community center**	Indoor attributes	How important is the size of the community center? (Likert scale: very important = 4)	3.34 (0.62)	1–4
		How important is the community center’s interior cleanliness? (Likert scale: very important = 4)	3.37 (0.57)	2–4
		How important are the time slots at the community center? (Likert scale: very important = 4)	3.40 (0.61)	1–4
		How important is convenience for using the community center? (Likert scale: very important = 4)	3.40 (0.62)	1–4
		How important it is for the community center to be safe for use by individuals with disabilities? (Likert scale: very important = 4)	3.33 (0.56)	1–4
	Outdoor attributes	How important is the design quality of the community center? (Likert scale: very important = 4)	0.36 (0.57)	2–4
		How important is the cleanliness around the community center? (Likert scale: very important = 4)	0.34 (0.59)	1–4
		How important is the safety around the community center? (Likert scale: very important = 4)	3.31 (0.62)	1–4
	Program attributes	How important is the quality of programs at the community center? (Likert scale: very important = 4)	3.27 (0.59)	1–4
		How important are the social interaction programs at the community centers? (Likert scale: very important = 4)	3.30 (0.61)	1–4
		How important are the education programs at the community centers? (Likert scale: very important = 4)	3.24 (0.59)	1–4
		How important are the leisure programs at the community centers? (Likert scale: very important = 4)	3.28 (0.61)	1–4
		How important are the childcare programs at the community centers? (Likert scale: very important = 4)	3.28 (0.65)	1–4
		How important is the housemaker supporting the programs at the community center? (Likert scale: very important = 4)	3.29 (0.60)	1–4
		How important are the free-sharing programs at the community centers? (Likert scale: very important = 4)	3.26 (0.58)	1–4
	Accessibility	How important is the accessibility to the community center from your home? (Likert scale: very important = 4)	3.43 (0.52)	2–4
**Satisfaction for community center**	Indoor attributes	How satisfied are you with the size of the community center? (Likert scale: very satisfied = 4)	3.14 (0.70)	1–4
		How satisfied are you with the community center’s interior cleanliness? (Likert scale: very satisfied = 4)	3.16 (0.65)	1–4
		How satisfied are you with the time slots offered by the community center? (Likert scale: very satisfied = 4)	3.05 (0.65)	1–4
		How satisfied are you with the convenience of use of the community center? (Likert scale: very satisfied = 4)	2.97 (0.66)	1–4
		How satisfied are you with the safety of people with disability and their use of the community center (Likert scale: very satisfied = 4)	2.98 (0.70)	1–4
	Outdoor attributes	How satisfied are you with the design quality of the community center? (Likert scale: very satisfied = 4)	3.21 (0.57)	2–4
		How satisfied are you with the cleanness around the community center? (Likert scale: very satisfied = 4)	3.01 (0.63)	1–4
		How satisfied are you with the safety around the community center? (Likert scale: very satisfied = 4)	3.03 (0.60)	1–4
	Program attributes	How satisfied are you with the quality of programs in the community center? (Likert scale: very satisfied = 4)	3.02 (0.52)	1–4
		How satisfied are you with the social interaction programs at the community center? (Likert scale: very satisfied = 4)	3.01 (0.57)	1–4
		How satisfied are you with the education programs at the community center? (Likert scale: very satisfied = 4)	3.04 (0.66)	1–4
		How satisfied are you with the leisure programs at the community center? (Likert scale: very satisfied = 4)	3.08 (0.68)	1–4
		How satisfied are you with the childcare programs at the community center? (Likert scale: very satisfied = 4)	2.96 (0.66)	1–4
		How satisfied are you with the housemaker supporting programs at the community center? (Likert scale: very satisfied = 4)	3.04 (0.59)	1–4
		How satisfied are you with the free-sharing programs at the community center? (Likert scale: very satisfied = 4)	3.00 (0.57)	1–4
	Accessibility	How satisfied are you with the accessibility of the community center from your home? (Likert scale: very satisfied = 4)	3.09 (0.39)	1–4
**Physical environments**	Walkable neighborhood	How satisfied are you with the walkability of your neighborhoods? (Likert scale: very satisfied = 4)	2.98 (0.60)	1–4
	Transit accessibility	How satisfied are you with transit accessibility in your neighborhoods? (Likert scale: very satisfied = 4)	2.85 (0.66)	1–4
	Amenity	How satisfied are you with the amenities in your neighborhoods? (Likert scale: very satisfied = 4)	2.97 (0.68)	1–4
	Crime safety	How satisfied are you with the crime and safety in your neighborhoods? (Likert scale: very satisfied = 4)	2.77 (0.61)	1–4
	Cleanliness	How satisfied are you with the cleanliness in your neighborhoods? (Likert scale: very satisfied = 4)	2.90 (0.39)	1–4

a SD: Standard deviation

b 1,000 KRW = about 0.76 USD as of 05/06/2023.

The socio-demographic characteristics encompass various factors, such as number of children, education level, age, household monthly income, number of household members, homeownership, gender, marital status, and region. Regarding residency duration and membership of the LCC, respondents were asked to provide the total time spent residing in the neighborhood and whether they were currently members of the LCC. Regarding the recognition and perceived importance of the community center, the respondents were asked whether they knew about its existence and whether they thought it was necessary. Regarding the physical environment, this study assessed the satisfaction levels of respondents regarding several factors, including walkability of the neighborhood, cleanliness, transit accessibility, availability of amenities, and crime and safety. The study employed a 4-point Likert scale to measure the satisfaction levels of the respondents, with 1 indicating “not satisfied” and 4 indicating “very satisfied.”

Additionally, the survey assessed the preferences of respondents regarding the importance of community centers in various aspects, including indoor attributes such as size, cleanliness, time availability, convenience, and safety; outdoor attributes such as design quality, cleanliness, and safety; program attributes such as program quality, interaction programs, education programs, leisure programs, childcare programs, household maker support programs, and free-sharing programs; and accessibility. Furthermore, respondents were asked about their level of agreement with the community center in the same aspects in terms of their preferences.

### Data analysis

To answer the first research question, cross-tabulation tables were generated to examine the difference between residents’ preferences and their satisfaction with access to the community center. For the second and third research questions, this study employed a structural equation model (SEM) to investigate the multifaceted factors related to community center usage frequency. Using the theoretical structure and factor loadings derived from factor analyses, the study constructed latent variables for community center preferences and satisfaction. A structural model was developed and the Maximum Likelihood (ML) approach was used to examine the proposed associations among all variables. Several indices were used to evaluate model fitness, including the Comparative Fit Index (CFI), Tucker-Lewis Index (TLI), and Root Mean Square Error of Approximation (RMSEA). An acceptable model had an RMSEA below 0.05 and CFI and TLI above 0.90 [34]. The analysis used the M-Plus 8.5 software to calculate the standardized coefficients, which were used to assess how each independent variable influenced the outcomes in comparison with others.

## Results

In terms of the results of descriptive statistics, the use frequency of the community center among respondents has a mean score of 1.67, with a standard deviation of 1.12, indicating some variation in the frequency of use. When examining socio-demographic characteristics, it is found that the average age of respondents is approximately 54.99 years, with a standard deviation of 10.59. This implies that the two selected neighborhoods consist of middle-aged residents, reflecting a relatively diverse age group. In terms of gender distribution, 26.76% of respondents are male. Regarding household monthly income, the mean income level is 7.56 on a scale that ranges from 1 (below 500,000 KRW) to 10 (4,500,000 KRW or more), showing a moderate-income range. Marital status data indicates that 1.54% of respondents are single. A significant majority, 53.86%, are homeowners. The education level of respondents averages at 2.89 on a scale from 1 (elementary) to 5 (graduate). On average, households consist of 3.17 members, with an average of 1.78 children.

Regarding living duration and involvement in the Local Community Committee (LCC), respondents have spent an average of approximately 165.46 months residing in the area, with a standard deviation of 88.04 months, indicating a wide range of residency durations. In terms of community committee involvement, 2.91% of respondents are currently members of the Local Community Committee (LCC), suggesting that a small proportion of the surveyed population are members of the committee.

In terms of recognition and the perceived necessity of the community center, the survey results reveal that a majority of respondents, specifically 66.72%, are aware of the existence of a community center in their town. This indicates a reasonably high level of recognition within the surveyed population. However, when asked about the necessity of the community center in their town, 43.34% of respondents expressed agreement, suggesting that a substantial portion of the population sees the community center as an essential asset to their community.

Respondents’ preferences for various attributes of the community center were assessed using a Likert scale, with a rating of 4 indicating that an attribute is “very important.” When evaluating indoor attributes, participants placed substantial importance on factors such as the size of the community center (mean rating of 3.34), interior cleanliness (mean rating of 3.37), the availability of suitable time slots (mean rating of 3.40), convenience in using the facility (mean rating of 3.40) and ensuring that the center is safe for individuals with disabilities (mean rating of 3.33). In contrast, outdoor attributes like the design quality of the community center (mean rating of 0.36) and the cleanliness of its surroundings (mean rating of 0.34) received notably lower importance ratings. Regarding program attributes, respondents indicated a significant emphasis on the quality of programs (mean rating of 3.27), social interaction programs (mean rating of 3.30), education programs (mean rating of 3.24), leisure programs (mean rating of 3.28), childcare programs (mean rating of 3.28), housemaker support for programs (mean rating of 3.29), and free-sharing programs (mean rating of 3.26). Additionally, respondents highly valued accessibility to the community center from their homes, giving it a mean importance rating of 3.43. These ratings provide insights into the aspects of the community center that residents consider most crucial.

Respondents’ satisfaction with different aspects of the community center was assessed using a Likert scale, with a rating of 4 indicating “very satisfied.” When it came to indoor attributes, respondents reported a moderate level of satisfaction with the size of the community center (mean rating of 3.14), interior cleanliness (mean rating of 3.16), and the time slots offered (mean rating of 3.05). However, they expressed lower levels of satisfaction with the convenience of using the community center (mean rating of 2.97) and the safety of individuals with disabilities using the facility (mean rating of 2.98). Regarding outdoor attributes, respondents reported a relatively high level of satisfaction with the design quality of the community center (mean rating of 3.21) and a moderate level of satisfaction with the cleanliness around the center (mean rating of 3.01) and safety in the vicinity (mean rating of 3.03). In terms of program attributes, satisfaction levels were generally moderate to high. Respondents expressed satisfaction with the quality of programs (mean rating of 3.02), social interaction programs (mean rating of 3.01), education programs (mean rating of 3.04), leisure programs (mean rating of 3.08), housemaker support for programs (mean rating of 3.04), and free-sharing programs (mean rating of 3.00). However, satisfaction with childcare programs was slightly lower (mean rating of 2.96). Respondents reported a relatively high level of satisfaction with the accessibility of the community center from their homes (mean rating of 3.09). These satisfaction ratings provide valuable insights into residents’ perceptions of the community center’s performance across various attributes.

The study evaluated residents’ satisfaction with various aspects of their neighborhoods using a Likert scale, with a rating of 4 indicating “very satisfied.” The findings revealed that, on average, residents reported moderate levels of satisfaction with neighborhood attributes. Regarding walkability, the mean satisfaction rating was 2.98, with a standard deviation of 0.60, indicating some variability in satisfaction levels. Similarly, transit accessibility had a mean satisfaction rating of 2.85 (standard deviation = 0.66), while amenities received a mean rating of 2.97 (standard deviation = 0.68). However, residents expressed slightly lower satisfaction with crime safety, with a mean rating of 2.77 (standard deviation = 0.61). In contrast, cleanliness in the neighborhoods garnered a mean satisfaction rating of 2.90 (standard deviation = 0.39). These statistics provide insights into residents’ varying levels of satisfaction with different neighborhood attributes, contributing to a comprehensive understanding of their living experiences.

In terms of the first research question regarding the difference of residents who prefer easy access to community centers and are satisfied with the actual access from their residence, Table 2 showed that 59.52% [(86+14)/168] of families had the same level of preference and satisfaction with the accessibility to community centers from their home locations. However, 36.9% [(3+59)/168] of the households were less satisfied with their actual accessibility to community centers than their stated preferences. This finding implies that the location of the community center may limit access for such residents, thereby decreasing the use of community centers.

**Table 2:** Cross-tabulation between the satisfaction and preference of accessibility to community center.

		Satisfaction of accessibility to community center		
		Somewhat satisfied	Satisfied	Very satisfied
**Preference of accessibility to community center**	Somewhat important	0.00% (0)	0.60% (1)	0.00% (0)
	Important	1.79% (3)	51.19% (86)	2.98% (5)
	Very important	0.00% (0)	35.12% (59)	8.33% (14)

Note: number of respondents in parenthesis

**Fig 3:**
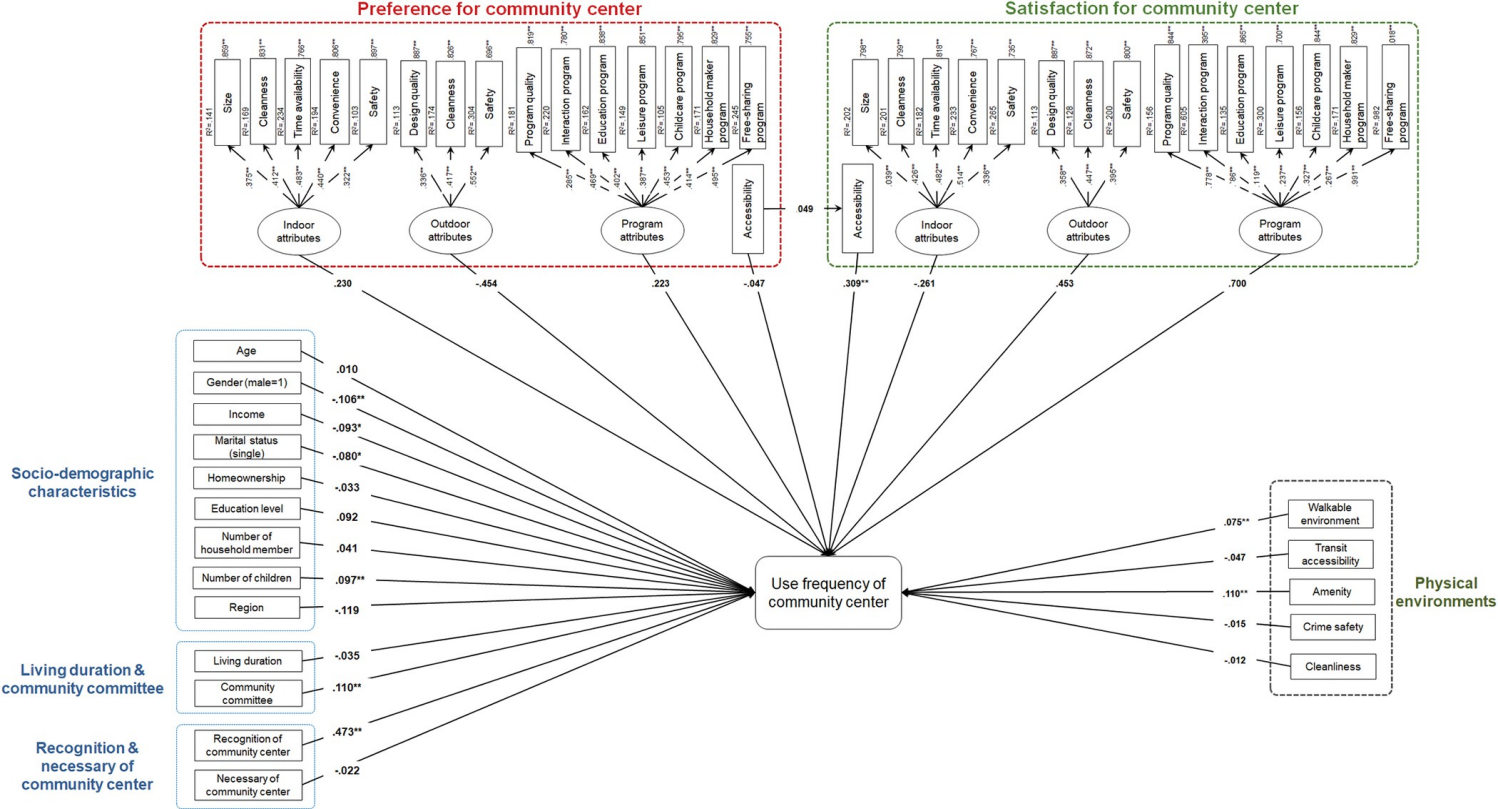
Model result.

The findings regarding the relationship between the frequency of community center usage and socio-demographic characteristics, duration of residence, community involvement, perception of the community center’s importance, physical environment, and preference for and satisfaction with the community center are depicted in Fig 3. The model’s goodness of fit was evaluated using RMSEA, which was 0.034, indicating a good fit as it was less than 0.05. The CFI was 0.93, which also indicated a good fit as it was greater than 0.90. Moreover, the TLI was 0.92, which is greater than 0.90, indicating a good fit.

For the second research question, “Does the preference for easy access to the community center have a direct or indirect (through actual access from their residence) influence on the frequency of use of the community center?,” the findings revealed that the preference for easy access to community centers had no significant impact on the frequency of use of community centers directly (-0.047) and indirectly through satisfaction with access to the center (0.049). However, satisfaction with easy access to the community center (0.309, *p* < 0.01) exhibited a significant positive relationship with the frequency of use of the community center. This indicates that the preference for accessibility to community centers was not a key factor affecting the frequency of use of community centers. However, those who were highly satisfied with their access to community centers were more likely to use them more frequently.

The third question was, “Does physical environment have a significant impact on the frequency of use of the community center after considering the preference for easy access to the community center?” The findings revealed that perceiving the environment as walkable (0.075, *p* < 0.01) was associated with a high frequent use of community centers. Moreover, the perception of a variety of amenities in the neighborhood was positively related to the increased use of community centers (0.110, *p* < 0.01).

Other variables were significantly correlated with the frequency of community center use. Regarding sociodemographic characteristics, males (-0.106, *p* < 0.01), high-income households (-0.093, *p* < 0.05), and those who were single (-0.080, *p* < 0.05) had a lower likelihood of using community centers, while households with more children (0.097, *p* < 0.01) showed a higher probability of using community centers. Moreover, individuals who were part of the community committee (0.110, *p* < 0.01) and those who knew of the existence of community centers in the town (0.473, *p* < 0.01) were more likely to use community centers.

## Discussion and conclusions

This study has provided crucial insights into the effect of preference for and satisfaction with accessibility to community centers and physical environments on the frequency of use of community centers. The findings of this study have various critical implications for future initiatives to promote community center use.

First, considering that some households are less satisfied with the actual accessibility to community centers, a needs assessment that considers transportation options and barriers would be beneficial for making community centers more accessible to all residents. The assessment explored the challenges in accessing community centers, such as insufficient non-motorized infrastructure, limited public transportation options, and a lack of parking facilities around the community center. The findings from the assessment could assist community leaders and stakeholders in determining transportation solutions, such as sidewalk or bike lane improvements or more public transportation options, to ensure that all residents have equitable access to community centers. Thus, it is crucial to conduct a needs assessment of community leaders and stakeholders to enhance accessibility to community centers for all residents.

Second, the finding that families with a higher level of satisfaction with proximity to community centers tended to use community centers more frequently suggests that initiatives designed to improve the utilization of community centers should focus on improving accessibility to community centers in the neighborhood to enhance user satisfaction. It also demonstrates that user satisfaction plays an important role in encouraging residents to use community centers more often. Promoting predetermined preferences for access to community centers may have limited outcomes in promoting their use. Based on this, even though the community center provides attractive activities or services, it is not easily accessible, and residents may have a lower probability of using the center because of their lower satisfaction with its proximity to the community center. Therefore, strategies to improve accessibility to community centers by enhancing user satisfaction, such as improving the physical infrastructure and nearby transportation options, should be prioritized.

Third, there might be gender-based differences in preference for activity participation, with males having less interest in community activities. High-income households may have greater access to private leisure activities and show less interest in community-based activities. Singles may prefer to engage in more independent leisure activities than community centers do. It is crucial for community organizers to account for differences in gender, income, and marital status when designing community center activities. Further exploration is required to determine whether these activities include males, high-income households, and single individuals. The use of community centers may increase if more activities are provided that are inclusive of different groups in the community. Households with more children may be more interested in community-related activities because they provide opportunities to socialize and engage with other families. Community leaders and organizers should promote family oriented activities to boost the utilization of community centers.

Fourth, participating in a community committee could potentially increase individuals’ sense of community and community belonging, resulting in a higher probability of using community centers as an approach to engaging neighbors and joining community activities. Being aware of community centers may increase individuals’ knowledge regarding resources and activities in the community, leading them to be more likely to use community centers. It is suggested that community leaders should put more effort into increasing the awareness of community centers and related resources and events offered to community residents through outreach programs and social media campaigns. By enhancing awareness, community residents tend to be more inclined to engage in events and use the community center more frequently, ultimately promoting social cohesion and community engagement.

Although this study provides important insights, it has some limitations that require attention. First, we caution that our findings may not be generalizable to other regeneration projects because this study only focused on two specific neighborhoods. Future research endeavors should aim to expand the cases of regenerated neighborhoods, encompassing various research areas in Korea as well as other countries. Second, the survey employed to evaluate individuals’ perceptions of the physical environment by relying on self-reporting, which may have introduced a recall bias. Objective measures of the physical environment can be used to address this issue and act as another method for assessing environmental factors. However, this study has limitations in employing objective measures to assess various environmental factors, primarily due to the small variation observed in the measures. The two selected neighborhoods were characterized by small-scale sites with limited variation in objective measures. Future study can extend the scope of the study by increasing the number of REIP cases and employing the objective measures. Third, this study focused solely on a restricted set of physical and environmental factors. Future studies should explore other aspects, such as the subjective perception of traffic safety, which comprises elements such as crosswalks and traffic-calming measures. Despite these limitations, the study has significant implications as it empirically identifies the nexus between multifaceted factors and community center usage frequency.

## Supporting information

S1 Data Analytical data set for the study. (XLSX)
